# Is round ligament varicosity in pregnancy a common precursor for the later development of inguinal hernias? The prospective analysis of 28 patients over 9 years

**DOI:** 10.1007/s10029-019-01928-6

**Published:** 2019-03-21

**Authors:** M. Lechner, R. Bittner, K. Borhanian, S. Mitterwallner, K. Emmanuel, F. Mayer

**Affiliations:** Department of Surgery, Paracelsus Medical University, Müllner Hauptstrasse 48, Salzburg, 5020 Austria

**Keywords:** Round ligament varicosity, Inguinal hernia, Pregnancy, Round ligament

## Abstract

**Purpose:**

Short-term effects of round ligament varicosity (RLV) in pregnancy have been investigated in small-scale studies. The long-term effects are unknown. This study aims to evaluate the risk of groin hernia manifestation after RLV in pregnancy, to delineate possible risk factors and to analyze the natural course of pregnancy and post-partum period with regard to RLV.

**Methods:**

In a prospective analysis 28 pregnant women with RLV presented to the hernia clinic over 9 years. After clinical and ultrasound examination during pregnancy and publication of early results in 2013 a second structured follow-up was conducted. Demographic data, hernia-specific risk factors, comorbidities, pregnancy and birth-related data as well as post-partum period were documented without loss of follow-up. In these women, all pregnancies that occurred, including the ones without RLV, were analyzed.

**Results:**

Median follow-up was 68 months (11.4–104.9). Only one groin hernia was found. No risk factors could be identified. After uncomplicated childbirth complaints subsided spontaneously in all but one patient within 4 weeks. Recurrence rates in subsequent pregnancies are up to 89%.

**Conclusion:**

Temporary RLV-induced dilation of the deep inguinal ring in pregnancy is not a common precursor for the development of inguinal hernias later in life. All findings support the theory that the hindrance of venous blood flow caused by the gravid uterus is an important contributing factor for RLV in pregnancy, which is self-limited but has a high risk of recurrence and is not an indication for surgery before or after delivery or for cesarean section.

## Introduction

Round ligament varicosity in pregnancy, caused by a distension of veins alongside the round ligament, has been described by various authors with a first report in 1962 [1]. It is an important differential diagnosis to groin hernias and yields a high rate of misdiagnosis [2–4]. It goes along with a temporary dilation of the deep inguinal ring [5].

Despite numerous publications on the topic, RLV remains a little known and hence rarely considered differential diagnosis of inguinal bulging during pregnancy within the medical professionals’ community. Publications are usually limited to case reports [2, 6, 7] and patient series with short follow-up periods. Potential risk factors, long-term consequences and in particular the possible manifestation of hernias in the area affected during pregnancy have not been analyzed to date and to our knowledge are evaluated in this study for the first time.

After childbirth the swelling usually subsides quickly in the majority of cases, but it is currently unknown if the temporary dilation of the deep inguinal ring during several months of pregnancy creates a potential weak spot in the abdominal wall that later in life gives rise to the development of groin hernias in these patients. The underlying cause of the condition and potential risk factors are unclear.

Therefore, it was the aim of this study to analyze long-term effects of RLV regarding development of inguinal hernias in pregnancy and to detect potential risk factors in the patient cohort followed.

## Materials and methods

From 12/2008 to 10/2017 all patients presenting to our department’s hernia clinic with RLV during pregnancy were included in a prospective analysis after clinical and ultrasound examination by two dedicated hernia surgeons. Several patients had more than one pregnancy. In some of these pregnancies, RLV was not present. All pregnancies of the women included were analyzed.

Early findings of 18 women were published in 2013 [5]. Thereafter the condition was observed in ten more women referred to our department’s hernia clinic until the end of the observational period. Overall 28 Caucasian patients and their medical course were evaluated with regard to long-term results of the condition.

The manifestation of inguinal hernias and the need for subsequent hernia repair during the follow-up period were defined as the primary endpoint of the study. Secondary endpoints were risk of recurrence of RLV in subsequent pregnancies and delineation of potential risk factors in the study population. With regard to the underlying mechanism of RLV manifestation the early findings of 2013 were re-evaluated in the larger cohort and could eventually be supported.

Demographic data, risk factors typical of hernia disease as far as present in the study cohort, pre-existing comorbidities, clinical signs and symptoms in pregnancy, family history of groin hernias, relevant information about pregnancy and child birth, course of RLV with regard to initial and subsequent pregnancies and births, post-partum course of complaints and rate of surgical interventions in the inguinal region were analyzed.

Median follow-up of 68 months (11.4–104.9) was complete at 100% and was performed by clinical and ultrasound examination in 54%.

Due to often large distances of patients’ homes to the clinic a structured telephone interview was conducted in 46%. The questions asked and the data retrieved were identical for all patients of the study. Valsalva’s maneuver with simultaneous self-examination during telephone interviews was carried out to provoke symptoms typical of possibly present groin hernias.

While demographic data, risk factors associated with inguinal hernia formation and comorbidities were documented upon initial presentation to the hernia clinic, pregnancy- and birth-related data and post-partum course were either retrieved by personal or telephone interview. The content of the questions asked is summarized in Tables 1, 2, 3 and 4. Complaints were defined as any abnormality in the groin noted by the patients at rest or Valsalva. For Valsalva’s maneuver patients were asked to press ‘as during childbirth’ in order to activate the abdominal press.

**Table 1:** Demographic data

	*n* or median	Range or %	Unit
Women examined (*n*)	28	–	–
Age	31.1	24.7–40.3	years
Height	167.5	154–178	cm
Weight	61	42–82	kg
BMI	20.8	16.6–32.0	kg/m^2^
Positive family history of inguinal hernias	5/28	17.90%	–
Pregnancies analyzed (*n*)	71	–	–
Pregnancies with complaints	46/71	64.80%	–
Pregnancies without complaints	25/71	35.20%	–
Pregnancies per woman	2	1–7	–
Births analyzed (*n*)^a^	66/71	–	–
Referral by	Gyn 22/28 (78.6%)	GP 3/28 (10.7%)	Self 3/28 (10.7%)

*BMI* body mass index: kg/m^2,^*Gyn* gynecologist, *GP* general practitioner, *Self* self-referral

^a^66 of 71 births were analyzed, the remaining 5 women were still pregnant at the end of data collection

**Table 2:** Comorbidities and hernia-specific risk factors

	*n*	%
Smoking	3/28	10.7
Gestational diabetes	1/28	3.6
Coagulation pathology	1/28	3.6
Vascular disease	0/28	0
Hemorrhoids in pregnancy	14/28	50
Varicose veins in pregnancy	13/28	46.4
Connective tissue disease	0/28	0

**Table 3:** Pregnancy-related data

	*n*	%
Onset by trimester		
1	2/28	7.1
2	25/28	89.3
3	1/28	3.6
Onset by pregnancy		
1	14/28	50
2	9/28	32.1
3	4/28	14.3
5	1/28	3.6
Side of body		
Right	16/28	57.1
Left	6/28	21.4
Bilateral	6/28	21.4

**Table 4:** Post-partum course

	*n*	%
RLV complaints subsided within 4 weeks	26/28	92.9
RLV again in ANY further pregnancy	16/18	89
RLV again in ALL further pregnancies	15/18	83
Complaints at end of follow-up	1/28	3.6
Follow-up over all	28/28	100
Follow-up by clinical and ultrasound examination	15/28	53.6
Follow-up by structured telephone interview	13/28	46.4
Hernias detected during follow-up	1/28	3.6
Undergone operations (1 for hernia, 1 for varicose veins)	2/28	7.1

## Results

**Fig. 1:**
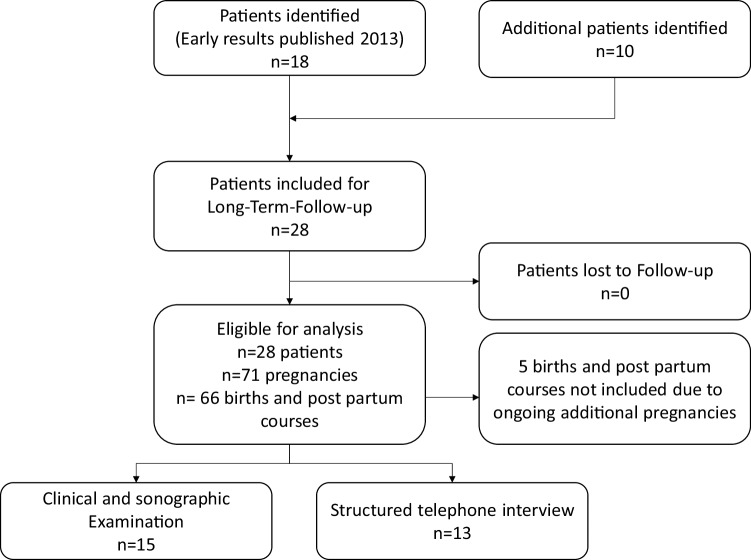
Flowchart describing study design with regard to recruitment of participants and follow-up

The flowchart of the study design is depicted in Fig. 1.

Only 1 patient developed a groin hernia in the post-partum course after a follow-up period of 60 months. In the analyzed 28 patients, no specific risk factors for the development of RLV during pregnancy could be delineated. Child’s weight at birth was analyzed and did not influence the manifestation of RLV. All women gave uncomplicated birth, mostly vaginal (55/66). The majority (79%) of patients was referred to our department by gynecologists for suspected groin hernias after clinical examination only. The diagnosis of RLV was then established by clinical examination and ultrasound in all 28 patients.

Detailed findings and results regarding demographic data, hernia-associated risk factors and co-morbidities, pregnancy-related data, and post-partum course of RLV are displayed in Tables 1, 2, 3 and 4.

## Discussion

Literature about the prevalence of groin hernias in women of any certain age is scarce.

One publication found it at 0.2% [8] for the age group described in this study. In comparison it therefore appears high at first sight with 4% in the present analysis. However, with only a single event in the study population these numbers cannot be compared directly. With just 1/28 patients affected, RLV in pregnancy cannot be understood as a common precursor for the development of inguinal hernias later in life.

A positive family history of first degree relatives diagnosed with inguinal hernias, BMI below 18.5 kg/m^2^, connective tissue disease and tobacco use have, among other parameters, been described as risk factors for the development of primary inguinal hernias in adults [9]. Six patients showed one of these risk factors. (Table 1).

With a diagnosed heterozygous factor V Leiden mutation, and a diagnosed post-thrombotic syndrome, one patient met the criteria for the risk factor coagulopathy syndrome without developing a hernia. Again, calculated at 4% of the study cohort this condition does not per se stand out as a risk factor for RLV. Of note she was the only study participant with ongoing tenderness, swelling and RLV symptoms at the end of the study period with a personal follow-up time of 98 months.

With the clearly isolated clinical discrepancy to the rest of the cohort and a diagnosed obstruction of her pelvic blood flow, this finding was seen as a hint towards pelvic outlet obstruction of the venous blood flow being one of the underlying mechanisms in the manifestation of RLV. Naturally this must be interpreted with caution and will need further verification from future observations.

In the present study cohort RLV occurred predominantly in the right groin, followed by left and bilateral manifestations (Table 3). Other authors found a higher prevalence on the left side [2, 7]. The reasons for these variable findings and their underlying cause remain unclear and warrant future exploration.

With regard to possible connective tissue pathology and vascular disease, hemorrhoids (50%) and varicose veins (46%) were common during pregnancy in the study population (Table 2). They were not overrepresented when compared to published literature about these conditions during pregnancy [10, 11] and with a relevant proportion of women not suffering from these conditions they were not understood to be directly associated with RLV either. The presence of these signs can therefore not be interpreted as specific risk factors for RLV, even though the manifestation in the second and third trimester may again point towards the influence of the gravid uterus on the pelvic blood flow with regard to the mechanism of manifestation. Soft-tissue disorders were not detected in any of the patients.

The children’s weight at birth should logically have an impact on the venous blood flow in the pelvis, with heavier and therefore bigger children leading to an increase in RLV in the mothers. However, birth weights were almost identical in patients with and without RLV and can therefore not be seen as a directly influencing factor. Another indicator for the possible presence of previously undetected soft-tissue weakness could have been a positive family history of hernias—which for the study purpose was defined as a history of any hernia in first degree relatives of the pregnant women. At first a rate of 5/28 patients (18%) appeared high, but given the high published lifetime risk of groin hernias of 27% in men and 3% in women [12] in the general population, it was found to be within normal range.

With inheritance being a risk factor for inguinal hernia formation, a higher number of women with positive family history should therefore lead to a higher prevalence of hernias in the cohort. This, however, was not the case.

With regard to long-term consequences we found that with the exception of the coagulopathic patient discussed above, complaints and swelling subsided spontaneously within 4 weeks after childbirth. This supports the results from a previous publication and is in line with other authors’ findings [2, 7]. Ongoing swelling and complaints for an extended period of time after delivery may therefore warrant further examination to rule out other potentially previously undetected causes in affected women.

Recurrence of RLV is very common and it is worth noting that the vast majority of affected women will face symptoms in some (89%) or even all (83%) of their further pregnancies.

At 83% the majority of pregnancies with (84%) and without (83%) RLV ended with vaginal deliveries. No complications occurred during childbirth. The same results have recently been published for women with diagnosed groin hernias in pregnancy [13]. Strong points of the study are the longest observational period for RLV in published literature, the complete follow-up and the comprehensive evaluation of potential risk factors, co-morbidities, pregnancy- and birth-related data.

Some limitations result from the size of the study cohort, which however comprises all patients with RLV referred to our hernia clinic over the course of 9 years. The need for follow-up by structured telephone interview arose in 46% of the cases because patients had moved after child birth or declared themselves too busy to attend the hernia clinic in the absence of complaints. Even though Valsalva’s maneuver was included in the telephone interview for self-examination, this might lead to a possible under-detection of asymptomatic hernias.

In conclusion RLV in pregnancy is not a common precursor of inguinal hernias. Specific risk factors for the condition could not be delineated in the study cohort. Despite being self-limited, recurrence of the condition is exceedingly common and must be expected in subsequent pregnancies. The underlying cause of RLV cannot ultimately be identified with the data available. The typical onset of symptoms during the second trimester of pregnancy and their usually spontaneous resolution shortly after childbirth supports the hypothesis that RLV is mainly the result of a hindrance of the venous blood flow in the pelvis caused by the mass of the gravid uterus. Persisting complaints in the patient with post-thrombotic syndrome of the pelvic veins support this thesis and warrant further exploration.

If after careful examination there is still an element of uncertainty about the presence of an additional undetected inguinal hernia, vaginal delivery can still be considered safe in patients with RLV and the presence of the condition should not lead to the indication of a cesarian section.

In view of the obtained referral statistics, further education of health care providers by distribution of these findings appears to be advisable.
